# Assessing the Efficiency of Antimicrobial Plant Extracts from *Artemisia afra* and *Eucalyptus globulus* as Coatings for Textiles

**DOI:** 10.3390/plants13040514

**Published:** 2024-02-13

**Authors:** Elvino Nortjie, Moses Basitere, Doice Moyo, Pardon Nyamukamba

**Affiliations:** 1 Bioresource Engineering Research Group, Department of Chemical Engineering, Cape Peninsula University of Technology, P.O. Box 1906, Bellville 7535, South Africa; elvinonortjie@gmail.com; 2 Academic Support Programme for Engineering in Cape Town (ASPECT), Centre for Higher Education Development, University of Cape Town, Rondebosch, Cape Town 7700, South Africa; 3 Technology Station Clothing and Textiles, Symphony Way, Bellville 7535, South Africa; moyod@cput.ac.za (D.M.); nyamukambap@cput.ac.za (P.N.)

**Keywords:** *Artemisia afra*, *Eucalyptus globulus*, antimicrobial textiles, phytochemical screening, minimum inhibitory concentration, zone of inhibition, washing durability

## Abstract

This study aimed to assess the antimicrobial activities of plant extracts from *Artemisia afra* and *Eucalyptus globulus* when used as coatings for textiles. A pulsed ultrasound-assisted extraction method (PUAE) was employed to obtain methanolic and hexanoic extracts from both plants. *Eucalyptus globulus* methanol extraction exhibited the highest yield at 22.76% (±0.61%), while *Artemisia afra* demonstrated lower yields. Phytochemical screening identified various secondary metabolites in the extracts, including phenols, quinones, and steroids. Antimicrobial tests against *Staphylococcus aureus* and *Escherichia coli* revealed varying degrees of susceptibility, with *Eucalyptus globulus* hexanoic extracts showing the highest activity against *Staphylococcus aureus* at an average percentage growth of 18.74% (±0.26%). Minimum inhibitory concentration (MIC) values were determined for the extracts, but complete inhibition did not occur at concentrations below 500 μg/mL. The extracts exhibited varying effects on *Staphylococcus aureus* and *Escherichia coli* growth, with some extracts promoting bacterial growth. Coating textiles with *Eucalyptus globulus* methanolic extracts demonstrated antibacterial activity against *Staphylococcus aureus* with the highest zone of inhibition observed in cotton-coated samples (258.4 mm^2^). Polyester-coated samples exhibited smaller inhibition zones, with the lowest observed in *Eucalyptus globulus* methanolic extract coating (65.97 mm^2^). Scanning electron microscope (SEM) analysis revealed visible surface morphology changes in coated fabrics, depicting fine, cluster, lumpy, flaky, and fragment-like morphologies. Laundering effects on coated fabrics were investigated, showing a significant decrease in antimicrobial activity after washing. Fourier-transform infrared spectroscopy (FTIR) identified functional groups in the extracts associated with antimicrobial properties. The complexity of the bioactive compounds suggests potential antimicrobial efficacy, resting on factors such as geographical location, climate, and extraction methods. Notwithstanding the limitations, this study contributes valuable insights into the use of plant extracts as antimicrobial coatings for textiles.

## 1. Introduction

The COVID-19 pandemic has shown us the importance of textiles in mitigating the transmission of harmful pathogens. The use of PPE, disinfectants, and social distancing was important for our safety as championed by the World Health Organization [1]. Textile coating advancements aim to enhance functional attributes such as durability, strength, and overall handling. Advancements in textile coatings have been directed towards increasing functional attributes, encompassing durability, strength, and overall handling. Other functional textile finishes may include flame retardancy, water repellency, anti-dirt, antibacterial, and antiviral properties [2].

Many synthetic fibres are more resistant to microbial attack than natural fibres. Organic fibres like cotton contain keratinous fibres and carbohydrates that serve as nutrients under optimal conditions, making them prone to microbial attack [3]. The global market for antimicrobial textiles manifests a steady growth trajectory, as projected by Fortune Business Insights (2021) with a compound annual growth rate of 5.3% from 2021 to 2028. The report attributes the growth of this market to the increasing demand for antimicrobial textiles in medical and apparel applications [4].

There is a wide range of natural to synthetic antimicrobial products on the market. Synthetic antimicrobial agents pose a great concern due to side effects and water pollution, hence the great demand for antimicrobial agents that fulfil the requirements of the regulating authorities [5]. Although herbal extracts have demonstrated effective antibacterial properties, the longevity of such coatings is inadequate when subjected to laundering [6]. Herbal extracts like *Artemisia afra* and *Eucalyptus globulus* have emerged as promising alternatives.

### 1.1. Artemisia afra

**Figure 1:**
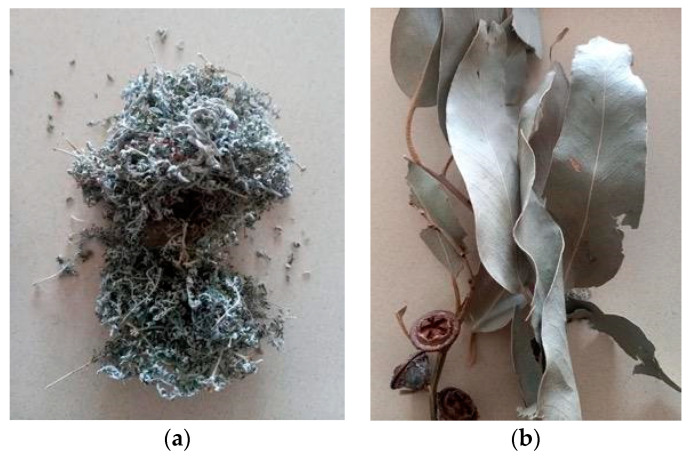
(**a**) *Artemisia afra* Jacq. and (**b**) *Eucalyptus globulus*.

*Artemisia afra* Jacq. Ex-Wild is a wormwood belonging to the Asteraceae family that is used for medicinal purposes in treating colds, flu, coughs, asthma, influenza, pneumonia, malaria, diabetes, earache, loss of appetite, and intestinal worms to malaria [7,8]. It is known as the African wormwood (English.); Wilde-Als (Afrikaans.); umhlonyane (isiXhosa); and umhlonyane (isiZulu). It is a soft aromatic shrub that grows up to 0.6–2 m (Figure 1a). It is one of the most popular medicinal plants in South Africa and is a common species with a wide distribution from the Cederberg Mountains in the Cape, northwards to tropical East Africa, and stretching as far north as Ethiopia [7].

The *Artemisia afra* species can be administered in various ways for personal medicinal use, such as enemas and infusions to cosmetics [9]. It is also used as a preservative in food [10] as well as an insecticide. The uses of *Artemisia afra* have been studied for its antimicrobial activities [7,11] and antifungal properties [7]. It was found to possess antifungal activity [12] and inhibitory activity against a broad spectrum of Gram-positive and Gram-negative bacteria [8,11].

### 1.2. Eucalyptus globulus

The Eucalyptus species are well-known plant species for their bioactive and pharmacological properties. The essential oil compounds of the genus *Eucalyptus* (Myrtaceae) have been well characterized [8]. *Eucalyptus globulus* is known to be the main furnisher of essential oils and is in high demand in the soap and cosmetic industries [13]. It is a tree that grows to a height of 60–80 m with a yellow-brownish smooth bark and sheds in long strips leaving a white-greyish colour on the surface of the bark [8]. Flowering occurs between May and January, and the flower buds appear in the leaf axils. The fruits are woody circular capsules that close at the edge in Figure 1b [7,8].

The Eucalyptus oil extracted from the leaf contains volatile terpenes and aromatic compounds and the most abundant monoterpenoid 1,8-cineole [7,8]. The British and European pharmacopoeia guidelines for using Eucalyptus oil for medicinal purposes state that it must contain at least 70% 1,8-cineole. The medicinal use of *Eucalyptus globulus* is based on its biological properties shown by the oils as antioxidant [14], anti-inflammatory, analgesic [15], and antimicrobial activities [7,8]. Clinical trials with Eucalyptus oil and the major component 1,8-cineole (eucalyptol) have been conducted to evaluate their efficacy in the treatment of a diverse range of conditions and diseases, including respiratory disorders [8] and head lice infestation [16]. The antimicrobial capabilities of essential oils have been reported for different species [8]. The *Eucalyptus globulus* leaf extract shows antibacterial activity against bacteria responsible for respiratory tract infections, such as *Staphylococcus aureus* ATCC 33591, *Streptococcus pyogenes* (*S. pyogenes*), and *Hemophilus influenzae* (*H. influenzae*).

Natural compounds have been extensively explored for new drug discoveries [17]. Extracts of different parts of plants contain bioactive compounds, such as alkaloids, steroids, tannins, glycosides, volatile oils, fixed oils, resins, phenols, terpenoids, and flavonoids that fight against diseases [18]. The phenolic phytochemicals from plants play a key role as antimicrobial agents [19,20].

*Artemisia afra* and *Eucalyptus globulus* plant species have been selected for this study as they may possess antimicrobial properties. Studies have shown that the mode of action of the antimicrobial agents on textiles can lead to agglomeration of the cell protein, damage the cell membrane, inhibit the cell membrane function, inhibit protein synthesis, and inhibit nucleic acid synthesis.

Antibiotic resistance to pathogenic bacteria has become a global problem, leading to natural-based antimicrobial compounds being studied extensively as an alternative therapeutic strategy to fight against microbial growth [8].

Antibiotics play a vital role in fighting bacterial infections. They also cause havoc in the healthcare and pharmaceutical sectors, accelerating socio-economic losses. Multidrug resistance is expected to increase by ten million deaths annually by 2050 [7]. Biological screening, separation of the phytochemicals, and clinical trials of medicinal plants have advanced over the years, unfolding the secrets of ancient herbal remedies [8]. Traditional medicine is effective in dealing with diseases caused by bacteria or oxidative stress [19,21,22]. Despite the announcement by the World Health Organization in the 1970s of *Artemisia afra* being unfit for human consumption, it is still used for ethnomedicinal purposes in South Africa today [23]. Eucalyptus species are the most represented species in the international pharmacopoeia in which *E. globulus* are the main supply of essential oils due to their medicinal properties [8]. This study investigates the potential of *Artemisia afra* and *Eucalyptus globulus* extracts as antimicrobial agents or textile coatings. The objective of this study is to contribute to the development of sustainable and effective solutions for impeding the dissemination of pathogenic agents. Natural-based antimicrobial compounds assume heightened importance as an alternative therapeutic strategy against microbial growth.

## 2. Materials and Methods

### 2.1. Materials

All solvents used for extraction were of analytical grade. Methanol AR 99.9% assay was purchased from Labchem (Pty) Ltd. (Cape Town, South Africa), while Hexane AR 98% assay and Ethanol AR 95% assay were purchased from Kimix Chemical and Lab Supplies CC (Cape Town, South Africa). Sodium hydroxide pellets 98% assay was purchased from North Side Diamond Tools (Johannesburg, South Africa) and Acetone AR 98% assay was purchased from Merck Millipore (Darmstadt, Germany). Sulfuric Acid 98% assay was purchased from Science World (Pty) Ltd. (Cape Town, South Africa), Acetic Acid AR Glacial 99% assay was purchased from Science World (Pty) Ltd. (Cape Town, South Africa), Chloroform AR 99% was purchased from Science World (Pty) Ltd., (Cape Town, South Africa), and Ferric Chloride 42.5% assay was purchased from Communica (Pty) Ltd. (Cape Town, South Africa). The commercial antibiotics Gentamicin and Ampicillin (Glentham, UK), *Staphylococcus aureus* ATCC 33591, and *Escherichia coli* ATCC 25922 from Anatech Instruments (Gauteng, South Africa), and the nutrient broth and tryptic soy broth Merk Millipore (Darmstadt, Germany) were also used.

### 2.2. Equipment

The following devices were used in the experiments: blender, Sonopuls Sonicator, Genevac MiVac Sample Evaporator, Perkin-Elmer FTIR spectrometer, incubator, oven, washing machine, padder, Perkin-Elmer Lambda-25 spectrophotometer, Tescan MIRA3 Scanning Electron Microscope, and ultrasonic bath.

### 2.3. Plant Material

#### 2.3.1. Sources of Plant Material and Fabric

The leaves of the medicinal plant *Artemisia afra*, except *Eucalyptus globulus*, were acquired from the South African National Biodiversity Institute located at Kirstenbosch Gardens. The biomass of *Eucalyptus globulus* was collected from Deer Park in Vredehoek, Cape Town. Both *Artemisia afra* and *Eucalyptus globulus* plant specimens were identified by Mr Pieter Winter at the South African National Botanical Institute (SANBI), with the assistance of Mr. Tielmann Haumann. The commercially available woven cotton fabric was obtained from SA Interlining Company, while the knitted polyester fabric was procured from Priontex Company with the assistance of Mr. Rainer Absenger.

#### 2.3.2. Plant Sample Preparation

The leaves of *Artemisia afra* and *Eucalyptus globulus* plants were harvested and thoroughly washed with distilled water to remove any dust particles. The leaves were then air-dried in the shade at room temperature for 30 days. Phytochemicals were extracted from the dried leaves using methanol and n-hexane solvents. The resulting extract was evaporated to determine the yield of the plant extractions.

#### 2.3.3. Plant Extraction

The solvent extracts were obtained using the pulsed ultrasound-assisted extraction (PUAE) method. Methanol and hexane were chosen based on their polarity. About 10 g of powdered leaves were extracted by sonicating in 100 mL of methanol and hexane. The PUAE was conducted for 20 min at 40 °C, at 50% amplitudes, with a pulse of 0.5 s on and 5 s off at a 20 kHz frequency with a probe diameter of 25 mm placed at a depth of 10 mm into the medium. Post-extraction, the mixtures were filtered through a Whatman LSC601 70 mm filter paper disc. The solvents were removed using a MiVac Sample Evaporator at 40 °C. The average extraction yield was calculated and reconstituted into its respective solvents and stored at −20 °C. Table 1 shows the extraction conditions used in this study.

#### 2.3.4. Preparation of Antimicrobial Cotton and Polyester Material

A total of 72 fabric cotton (148 g per square meter) and polyester fabric (125 g per square meter) samples were prepared and cut into squares measuring 3 cm × 3 cm. Afterwards, the fabric cuttings were washed with a mixture of ethanol and water using ultrasonication for 30 min to eliminate any wax and impurities on the fabric surface. Next, the fabric cuttings were dried for an hour at 80 °C in a warm air oven. Plastic containers were then used to immerse the fabric cuttings in the chosen extracts. The cuttings were left to soak for 24 h at 25 °C in an FHM incubator shaker and then dried in a warm air oven at 80 °C for one hour to eliminate the solvent. Finally, the fabric cuttings were utilized to determine the zone of inhibition against *S. aureus* ATCC 33591 and *E. coli* ATCC 25922.

### 2.4. Experimental Method and Procedure

**Figure 2:**
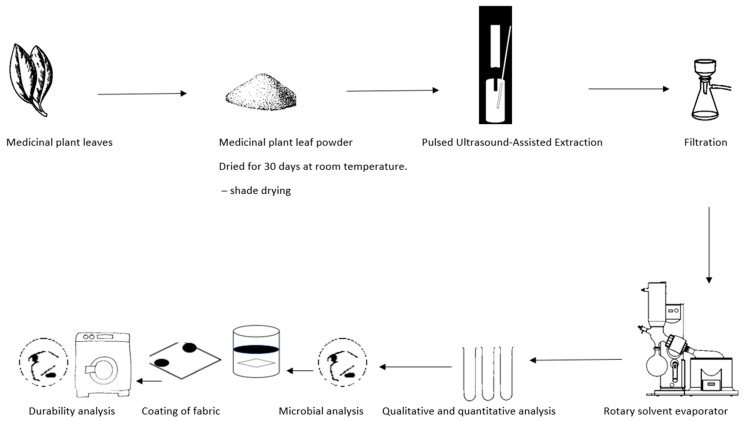
Process flow diagram of the experimental setup of the antimicrobial textiles. Experiments were carried out to determine various factors related to the effectiveness of coated fabrics in preventing microbial growth. Specifically, the minimum inhibitory concentration (MIC), the zone of inhibition (ZOI), and the durability of the coated fabric after undergoing a laundering process according to ISO 6330:2012 [24]. were analysed. The antimicrobial effectiveness of the coated fabric was then evaluated by measuring the ZOI against the selected strains *S. aureus* ATCC 33591 and *E. coli* ATCC 25922, as well as appropriate controls.

The process flow diagram of this study is shown in Figure 2 below.

### 2.5. Characterizations

The surface morphology of both uncoated and coated cotton and polyester fabrics was examined before and after coating of fabrics using a Tescan MIRA3 Scanning Electron Microscope from the Tescan Group, a.s. (CZ). This analysis involved studying the fabric’s surface at two different magnification levels: a low magnification of 1000× and a high magnification of 25,000×. The specimens were subjected to analysis under an applied voltage of 5 KV. The functional groups present in the woven cotton and knitted polyester fabric were analysed before and after the application of an antimicrobial finish. Fourier-transform infrared spectroscopy (FTIR) was used to perform the analysis, using a Cary 660 spectrometer from Agilent Technologies (Columbia, SC, USA) with a wavenumber range of 400–4000 cm^−1^.

#### 2.5.1. Determination of Minimum Inhibitory Concentration

The nutrient and tryptic soy broth growth media for the selected test strains, *Staphylococcus aureus* ATCC 33,591 and *Escherichia coli* ATCC 25922, respectively, were autoclaved for 25 min at 120 °C and under pressure. Once cooled, the liquid media were inoculated with agar plate cultures. The liquid cultures were incubated at 37 °C at 160 rpm for 24 h. The next day, the cultures were analysed by Gram staining to ensure no contamination. The optical density (OD) of the cultures was determined at a wavelength of 600 nm using a Perkin-Elmer Lambda-25 spectrophotometer and diluted to an OD600 = 0.2. The extracts were serially diluted in the range of 5–500 μg and 50 μL of each sample was pipetted into a sterile 96-well plate in triplicate, and the plate was sealed with a Breath-Easy seal to allow for respiration. The plates were incubated at 37 °C for 24 h. Following that, 20 μL of 0.25% (*w*/*v*) MTT solution (in phosphate-buffered saline) was added to each experimental well and incubated for 4 h at 37 °C. The reaction was stopped by adding 100 μL of 100% DMSO, and the OD was determined using a microtiter plate reader. To minimize errors, each sample was evaluated in its 96-well plate: for 12 samples, 24 plates of 96-well plates (two test strains) were used. The minimum inhibitory concentration (MIC) is determined as the lowest concentration at which total inhibition of the test strain occurs. Controls included wells with media and test strains without any additions.

#### 2.5.2. Determination of the Inhibition Zone

*Step 1: Preparation of liquid cultures*

Specific liquid growth media (5 mL per 50 mL glass flask; five flasks per strain) were prepared for each test strain:
*Staphylococcus aureus* ATCC 33591Nutrient broth*Escherichia coli* ATCC 25922Tryptic soy broth

The cultures were analysed by Gram staining to ensure that the cultures were not contaminated. The optical density of the cultures was determined at 600 nm using a Perkin-Elmer Lambda-25 spectrophotometer and diluted to an OD_600_ = 0.2. These diluted cultures were used in the agar diffusion test [25].

*Step 2: Preparation of agar plates*

A total of twelve samples, twelve agar plates per test strain, were prepared. This required the preparation of the following (also including plates for controls):

25 tryptic soy agar plates = 500 mL of media

25 nutrient agar plates = 500 mL of media

Each sample was assessed in triplicate on each plate and was prepared as follows:

For each agar plate, 100 μL of the OD600 = 0.2 cultures were spread-plated onto the agar and allowed to diffuse into the agar. Each textile specimen was pre-treated with UV (30 min on each side) to kill off any contaminants and then placed onto the agar plate containing the test strain (to ensure that the UV treatment was effective, the material was also placed on agar containing no test strain). For each test strain, a commercial antibiotic was used as a positive control. Gentamicin and ampicillin were used for the bacterial test strains. Samples were plated on agar plates and incubated at 37 °C overnight. The zones of inhibition were measured to determine whether growth inhibition occurred as reported by Marković et al. [25].

### 2.6. Durability Tests

The purpose of this durability test is to evaluate the fabric’s resistance to degradation and the loss of antimicrobial effectiveness. The zone of inhibition test assesses the effectiveness of the antimicrobial coating, helping to determine the bacteria’s resistance against antimicrobial agents. The durability of the extract coating applied to textiles under simulated home laundering conditions was assessed using the washing method, ISO 6330:2012 [24]. The coated fabric samples were cut into 3 × 3 cm squares and weighed to determine the amount of washing powder and water required for laundering. The fabric samples were washed at 60 °C using the Pyrotec MB2 washing machine, rinsed in cold water, and dried in a hot air oven at 80 °C for 1 h. Each textile sample was pre-treated with UV for 30 min on each side to kill off contaminants and then placed onto the agar plate containing the test strain. The material was also placed on agar containing no test strain to ensure that the UV treatment was effective. The agar plates were incubated at 37 °C overnight, and the zones of inhibition were measured.

### 2.7. Phytochemical Screening of Artemisia afra and Eucalyptus globulus

The extracts were subjected to phytochemical screening of bioactive compounds. The methanolic and hexanoic extracts of the leaves were screened for the presence of phenols, flavonoids, quinones, tannins, saponins, terpenoids, and steroids. The quantitative results are expressed as (+) for positive and (−) for negative results.

#### 2.7.1. Detection of Phenols

The extract of 1.0 mL was diluted in distilled water to 3.0 mL followed by filtration. Four drops of 5% iron (III) chloride solution were added to the solution. The observation of a dark green colour indicated the presence of phenol [7].

#### 2.7.2. Detection of Flavonoids

The extract of 1.0 mL was transferred into a test tube and treated with four drops of sodium hydroxide solution. The observation of a yellow colour indicated the presence of flavonoids [7].

#### 2.7.3. Detection of Quinones

The extract of 1.0 mL was placed in a test tube and mixed with 1.0 mL of pure 98% sulphuric acid. The observation of a red colour indicated the presence of quinones [7].

#### 2.7.4. Detection of Tannins

An approximately 1 mL aliquot of the extract was transferred into a 5 mL test tube followed by the addition of three drops of 5% iron (III) chloride, and the observation of a greenish-black precipitate indicated the presence of tannins [7].

#### 2.7.5. Test for Saponins

An approximately 0.5 mg aliquot of the extract was mixed vigorously with 5 mL of distilled water. The formation of stable foam indicated the presence of saponins [7].

#### 2.7.6. Test for Steroids

Approximately 1.0 mL of extract was shaken with 99% chloroform, and to the chloroform layer, 98% sulphuric acid was added slowly by the sides of the test tube. The observation of a red colour indicated the presence of steroids [7].

#### 2.7.7. Test for Terpenoids

An approximately 0.5 mL aliquot of the extract was mixed with 2.0 mL of 99% chloroform followed by the addition of 3.0 mL of concentrated 98% sulphuric acid to form a layer. The observation of a reddish-brown colour indicated the presence of terpenoids [26].

## 3. Results and Discussion

### 3.1. Extraction Yield

The average extract yields of methanolic and hexanoic *Artemisia afra* and *Eucalyptus globulus* extracts are summarized in Table 2. All experiments were conducted in triplicate.

The average yields of methanolic and hexanoic extracts from *Artemisia afra* and *Eucalyptus globulus* are summarised in Table 2. All experiments were conducted in triplicate. The extraction yields of *A. afra* and *E. globulus* were investigated by studying the effects of various solvents, including methanol and hexane. The findings demonstrated that the extraction yields differed depending on the solvent used, attributed to variations in solvent polarity. The yield of extraction is a measure of solvent effectiveness in extracting phytochemicals from the material under selected conditions. The results in Table 3 show that the maximum yield was obtained with *E. globulus* methanol extraction at 22.76% (±0.61%), followed by hexane extraction at 3.98% (±0.06%). *A. afra* methanol extraction yielded 9.22% (±0.40%), followed by hexane extraction of 2.19% (±0.28%). Higher yields were observed in the methanolic extracts of *A. afra* and *E. globulus* compared with the hexanoic extracts. Notably, *A. afra* produced the lowest yield. Overall, the yields of methanolic and hexanoic extracts differed significantly. The improved extraction yields could be attributed to cavitation effects caused by high-intensity ultrasound and solvent polarity.

### 3.2. Phytochemical Screening Analysis

Plant parts contain bioactive compounds with various phytochemical compounds, including alkaloids, flavonoids, steroids, terpenoids, tannins, saponins, phenols, and quinones, all of which possess antimicrobial properties. In this study, the crude extracts were screened for the presence of phenols, flavonoids, quinones, tannins, saponins, terpenoids, and steroids. The phytochemical screening of *Artemisia afra* and *Eucalyptus globulus* crude leaf extracts is shown in Table 3, and it revealed the presence of different secondary metabolites, such as phenols, quinones, and steroids. Tannins, saponins, and terpenoids were not found in the hexanoic extracts of *Artemisia afra.* Flavonoids were absent in the methanolic extraction of *Eucalyptus globulus.* Tannins and saponins were also not found in the hexanoic extraction of *Eucalyptus globulus*.

Phytochemical screening revealed the presence and absence of different secondary metabolic compounds, such as phenols, quinones, terpenoids, flavonoids, saponins, steroids, and tannins. This could be attributed to the different plants, plant parts, types of solvents used for extraction, and geographical areas exhibiting climatic conditions that determine their ability to support rain-fed cultivation [27].

Phytochemical screening of crude leaf extracts of *A. afra* and *E. globulus* revealed that both plants had phenols, quinones, and steroids. The phytochemical screening indicates that the methanol extract produced positive results for the analysed phytochemicals. Many solvent extractions have been performed to obtain phytochemical compounds for use against pathogens. From the phytochemical assessment, phenols were found in all the extractions. The mechanism of action of polyphenols is to bind to adhesins, inhibit enzyme-substrate deprivation, complex with the cell wall, and disrupt the membrane and metal ion complexation [28]. Quinones were also found in all the extractions. The mechanisms of action of quinones include inactivating enzymes, complexing with the cell wall, and binding to adhesins [28]. Tannins were present in the methanolic extracts, as shown in Table 3. Tannins allow the binding of adhesins, inhibit enzyme-substrate deprivation, complex with the cell wall, and form metal ion complexes [28,29]. Flavonoids were present in most of the extracts except for the *Eucalyptus globulus* methanolic extracts. Flavonoids form a complex with the cell wall and bind to adhesins. The extracts were also screened for terpenoids which disrupt the membrane wall. The extracts were screened for saponins, and they were only present in the methanolic extracts. The mechanism of action of saponins inhibits the growth of Gram-positive bacteria and Gram-negative bacteria by disrupting cell membranes [28]. The extracts were screened for steroids, and they were found in all the extracts. Steroids derived from medicinal plants are known to possess antibacterial and insecticidal properties [30].

### 3.3. Antibacterial Activity of Plant Extracts

**Figure 3:**
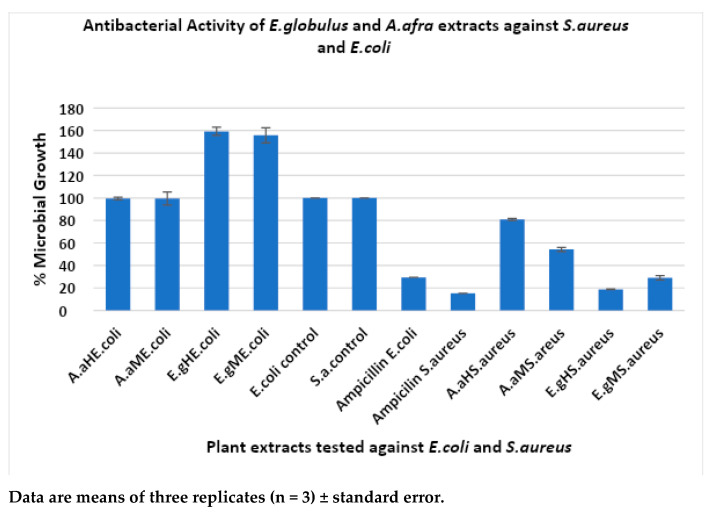
Antibacterial activity of plant extracts *A. afra* and *E. globulus* against *S. aureus* and *E. coli*. A.a: *Artemisia afra*; E.g.: *Eucalyptus globulus*; H: hexane; and M: methanol.

The antibacterial activity of plant extracts *A. afra* and *E. globulus* was investigated against Gram-positive bacteria, *Staphylococcus aureus* ATCC 33591, and Gram-negative bacteria, *Escherichia coli* ATCC 25922, and the results are shown in Figure 3.

The hexanoic *E. globulus* extracts showed good results in suppressing microbial growth of *S. aureus* ATCC 33591 at an average percentage of 18.74% (±0.26%) growth due to a higher number of phytochemicals like polyphenolic compounds being present, which are known to have antimicrobial activity, followed by the methanolic *E. globulus* extraction at an average of 29.03% (±1.03%) growth. The *A. afra* methanolic extractions showed antibacterial activity against *S. aureus* at an average of 54.23% (±1.74%), and the hexanoic *A. afra* extracts showed an average percentage growth of 80.87% (±0.87%) and exhibited the least activity against *S. aureus* ATCC 33591 strains at 250 µg/mL. The *S. aureus* microbial growth was observed to be more susceptible to the *E. globulus* hexanoic extracts, followed by the *E. globulus* methanolic extracts compared with the *A. afra* methanolic and hexanoic extracts. As expected, the control showed no antimicrobial activity (Figure 3). Ampicillin shows antimicrobial activity against *S. aureus* ATCC 33591 at an average growth of 15.27% as expected. The evaluation of the *A. afra* and *E. globulus* extracts against *E. coli* ATCC 25922 was the least susceptible compared with *S. aureus*. The methanolic *A. afra* showed slight activity against *E. coli* ATCC 25922 at an average percentage growth of 99.52% (±5.87%), followed by the hexanoic *A. afra* extracts at 99.52% (±1.17%). The methanolic and hexane *E. globulus* extracts showed no effect against Gram-negative *E. coli* ATCC 25922. Instead, they showed enhanced growth of an average of 159.25% (±3.65%) and 155.70% (±6.70%) at 250 µg/mL, respectively. This could be due to compounds from plants that enhance bacterial growth. As expected, the control sample did not show any antimicrobial activity. Ampicillin shows antimicrobial activity against *E. coli* ATCC 25922 at an average growth of 29.34%. The results show that *E. coli* ATCC 25922 was the most resistant strain against the plant extracts. *S. aureus* ATCC 33591 was the most susceptible strain to the plant extracts.

### 3.4. Minimum Inhibition Concentration Analysis

The MIC, which stands for minimum inhibitory concentration, is defined as the lowest concentration of the extract that can prevent the growth of microorganisms. To test the extract’s effectiveness against different strains of bacteria, specific liquid growth media were prepared for each test strain in 50 mL glass flasks at a volume of 5 mL per flask, with three flasks per strain. The results showed that only some of the samples displayed activity in the liquid media against the tested ATCC bacterial strains, as detailed in Table 4.

This study evaluated the MIC values of methanolic and n-hexane extracts of *A. afra* and *E. globulus* leaf extracts against *S. aureus* and *E. coli*. Although some bioactivity was observed, complete inhibition of the cultures did not occur, and all MIC values were above 500 μg/mL. The antimicrobial tests yielded MIC values ranging from 5 μg/mL to above 500 μg/mL, as shown in Table 4. The methanol extract of *A. afra* exhibited sensitivity against the *S. aureus* ATCC33591 strain, with a MIC value ranging from 5 μg/mL to 25 μg/mL. However, some extracts, such as *E. globulus* hexanoic extract against *S. aureus* and *E. globulus* methanolic extract against *E. coli* ATCC 25922, did not show any minimum inhibition concentration. Bacterial culture growth exceeded 100% during sample analysis, possibly due to plant compounds that promote bacterial growth. Samples that exhibited antimicrobial activity in liquid-based assays did not show bioactivity in agar-based assays [25].

### 3.5. Zone of Inhibition Analysis

The zone of inhibition analysis (ZOI) was conducted against both bacterial types *S. aureus* and *E. coli*. The antimicrobial fabric was produced by using the *A. afra* and *E. globulus* methanolic and hexanoic extracts via an immersion process for 24 h. The green polyester and light blue cotton fabric per sample were used for the zone of inhibition analysis. The fabrics were cut into 2 cm diameter circles and subjected to the above protocol. Only some samples exhibited bioactivity as shown in Table 5.

**Figure 4:**
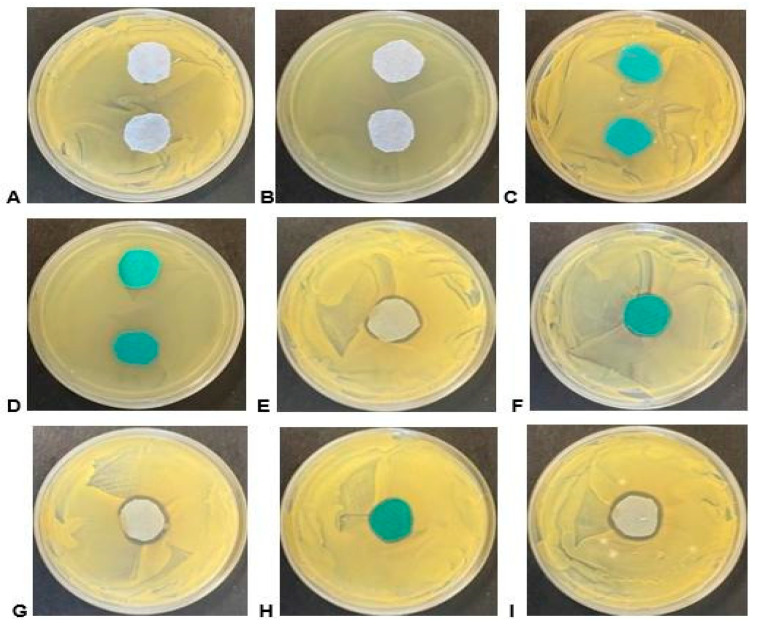
Bioactivity of material samples on agar plates containing the ATCC test strains. (**A**) Light blue/grey cotton material control sample on *S. aureus*; (**B**) light blue/grey cotton material control sample on *E. coli*; (**C**) turquoise polyester material control sample on *S. aureus*; (**D**) turquoise polyester material control sample on *E. coli*; (**E**) sample 10 light blue/grey cotton material on *S. aureus*; (**F**) sample 10 turquoise polyester material on *S. aureus*; (**G**) sample 11 light blue/grey cotton material on *S. aureus*; (**H**) sample 11 turquoise polyester material on *S. aureus*; and (**I**) sample 12 light blue/grey cotton material on *S. aureus*.

Moist environments promote bacterial growth, and textiles often serve as a breeding ground for bacteria. To assess the antibacterial properties of fabrics, the coated and uncoated polyester and cotton samples against Gram-positive *S. aureus* and Gram-negative *E. coli* bacteria were examined. As indicated in Table 5 only samples 10 to 12 exhibited inhibition zones. Cotton fabrics, which contain cellulose polymer, facilitate bacterial adherence, whereas polyester fabrics, with their polymer backbone, are less susceptible to bacterial attachment. However, applying *Eucalyptus globulus* methanolic extracts onto fabrics altered the fabric’s surface properties. Our study revealed that samples 10 to 12, which were coated with the extract, displayed antibacterial activity, as illustrated in Figure 4 by the presence of zones of inhibition around the material.

Figure 4 shows that the coated fabric demonstrated a larger zone of inhibition compared with the untreated samples. Among the coated fabrics, the highest zone of inhibition was observed in cotton coated with *E. globulus* methanolic extract (258.4 mm^2^) against *S. aureus,* while the smallest zone of inhibition was observed in polyester coated with *E. globulus* methanolic extract (65.97 mm^2^) as depicted in Figure F, sample 10 (turquoise material), on *S. aureus*. The bioactivity was only evident against the *S. aureus* strain. Although some samples displayed activity in liquid media assays, they failed to exhibit bioactivity in the agar-based assay; the inactivation of active compounds during the treatment or preparation of the material samples or the enhanced absorption of other active compounds, when applied to the material, could be the potential reasons for this result. The bioactivity seen in such assays also relies on the diffusibility of compounds from the material into the agar medium, leading to the inhibition of growth. It is essential to note that bacterial growth is rampant in humid conditions [25]. Natural fibres like cotton are more susceptible to microbial attack because they easily retain water. In contrast, the microbial attack is slower in synthetic fibres such as polyester fabric due to their polymer backbone [31].

### 3.6. SEM Analysis

**Figure 5:**
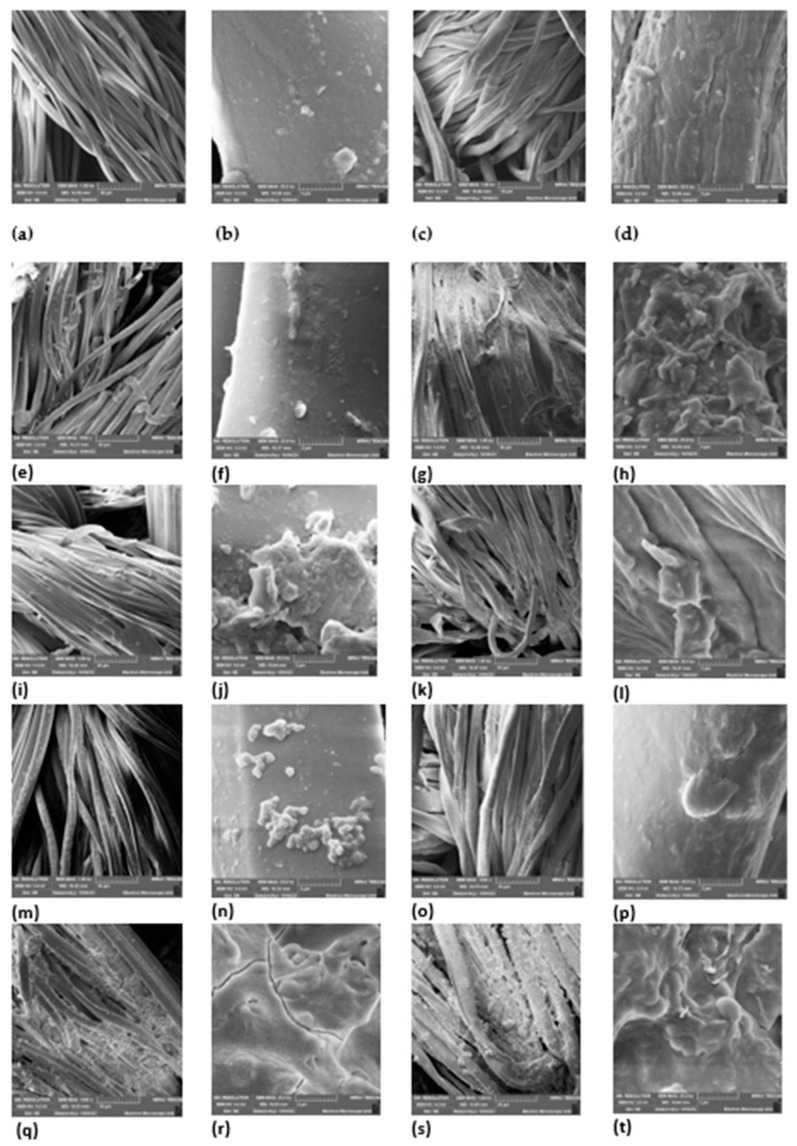
SEM micrographs of (**a**) uncoated polyester 1000× magnification, (**b**) uncoated polyester fabric 25,000× magnification, (**c**) uncoated cotton fabric 1000× magnification, (**d**) uncoated cotton fabric 25,000× magnification, (**e**) coated *A. afra* hexane polyester fabric 1000× magnification, (**f**) coated *A. afra* hexane polyester fabric 25,000× magnification, (**g**) coated *A. afra* hexane cotton fabric 1000× magnification, (**h**) coated *A. afra* hexane cotton fabric 25,000× magnification, (**i**) coated *A. afra* methanol polyester fabric 1000× magnification, (**j**) coated *A. afra* methanol polyester fabric 25,000× magnification, (**k**) coated *A. afra* methanol cotton fabric 1000× magnification, (**l**) coated *A. afra* methanol cotton fabric 25,000× magnification, (**m**) coated *E. globulus* hexane polyester fabric 1000× magnification, (**n**) coated *E. globulus* hexane polyester fabric 25,000× magnification, (**o**) coated *E. globulus* hexane cotton fabric 1000× magnification, (**p**) coated *E. globulus* hexane cotton fabric 25,000× magnification, (**q**) coated *E. globulus* methanol polyester fabric 1000× magnification, (**r**) coated *E. globulus* methanol polyester fabric 25,000× magnification, (**s**) coated *E. globulus* methanol cotton fabric 1000× magnification, and (**t**) coated *E. globulus* methanol cotton fabric 25,000× magnification.

The surface morphologies of both uncoated and coated cotton and polyester fabrics were examined through the utilization of a scanning electron microscope (SEM). This analysis involved studying the fabric’s surface at two different magnification levels: a low magnification of 1000× and a high magnification of 25,000×. The specimens were subjected to analysis under an applied voltage of 5 KV as shown in Figure 5.

Figure 5 displays the SEM images of both uncoated fabric and fabric coated with a plant extract. The SEM micrographs (a)–(d) of the uncoated fabric reveal smooth longitudinal filaments of the fibres, with no distinguishable particles on their surfaces. Micrographs (e)–(h) reveal a flaky appearance on the fabric as well and in micrographs (i)–(l), a distinguishable distribution of particles can be seen on the fabric. Micrographs (m) and (n) reveal a cluster-like appearance of particles on the fabric whereas micrographs (o) and (p) show a more lump-like appearance of particles on the fabric. Micrographs (q)–(t) reveal a flaky-like appearance on the fabric. Overall, micrographs (e)–(t) reveal clear particles visible on the fabric surfaces, which depict surface coverage characterized by the fine, cluster, lumpy, flaky, and fragment-like morphology formed on the coated fabric. Textured surfaces are apparent, resulting from deposits on both polyester and cotton fabrics. The extracts strongly penetrated the surface of the fabrics and confirmed deposition on the fabric.

### 3.7. Durability to Washing

Frequent washing of coated fabrics may affect the durability of the coating on the fabric as it plays a key role in coated textiles. This study also focuses on the effect of laundering on the coated textiles against the selected bacterial strains. The ZOI of the microorganisms were measured to determine whether growth inhibition occurred.

After laundering, no activity was observed against *S. aureus* and *E. coli*. This could be due to the inactivation of active compounds during sample treatment or preparation, or the improved bioavailability of other active compounds when applied to the material. On agar plates containing ATCC test strains, no bioactivity was observed on the material samples after laundering. The antimicrobial activity of the samples that had previously shown activity against the selected bacterial strains decreased significantly after washing. The inactivity of the coated fabrics may be due to factors such as reduced extract concentration, variation in phytochemicals per extract, solvent polarity, and the coating technique used to apply the extracts onto the fabrics.

**Figure 6:**
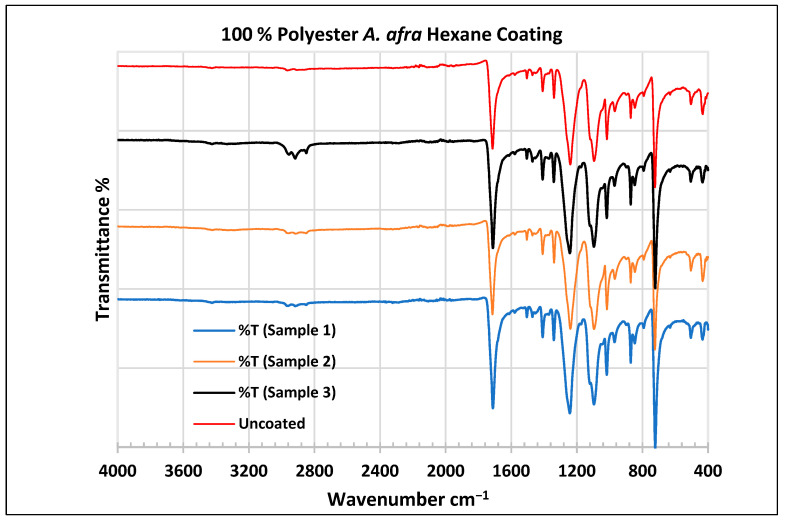
FTIR spectra of polyester fabrics uncoated (U) and coated (1–3) with *A. afra* hexane coating. Samples 1–12 were performed in triplicate.

**Figure 7:**
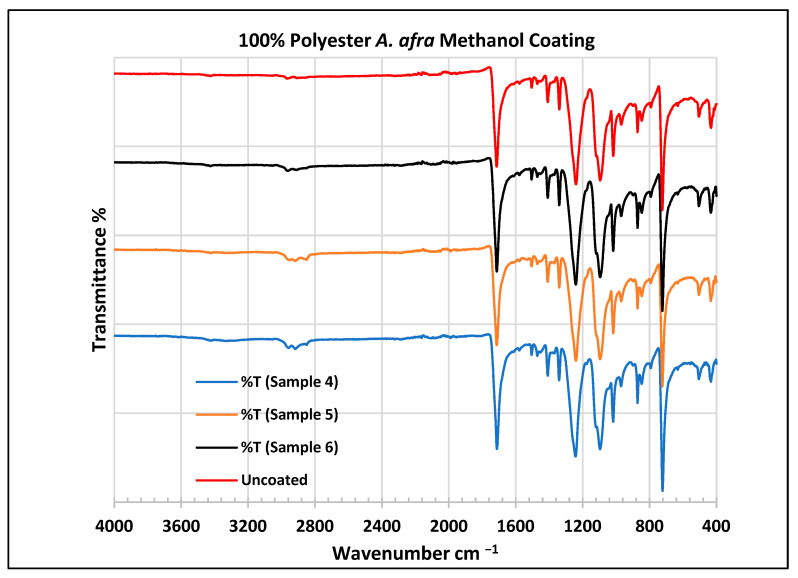
FTIR spectra of polyester fabrics uncoated (U) and coated (4–6) with *A. afra* methanol coating.

**Figure 8:**
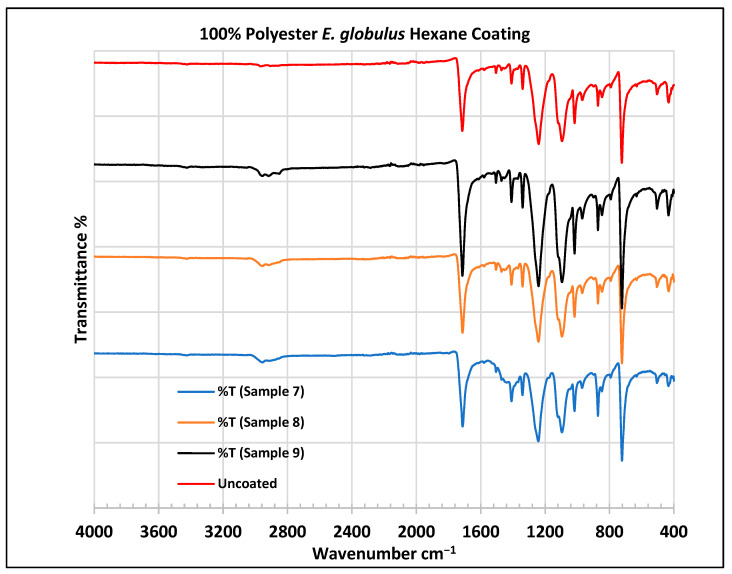
FTIR spectra of polyester fabrics uncoated (U) and coated (7–9) with *E. globulus* hexane coating.

**Figure 9:**
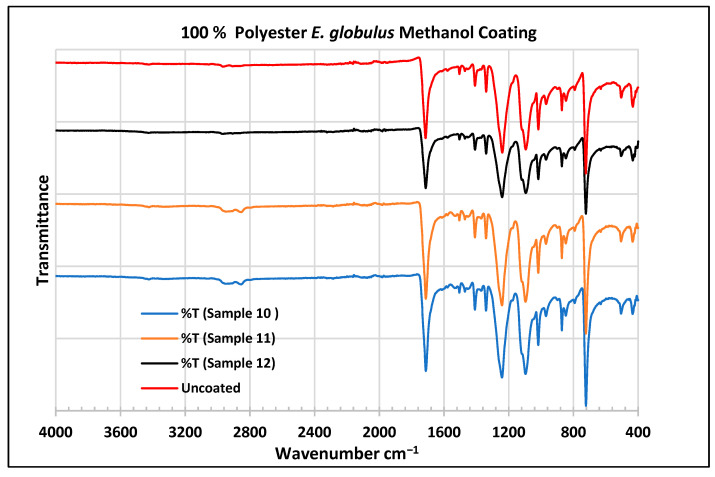
FTIR spectra of polyester fabrics uncoated (U) and coated (10–12) with *E. globulus* methanol coating.

**Figure 10:**
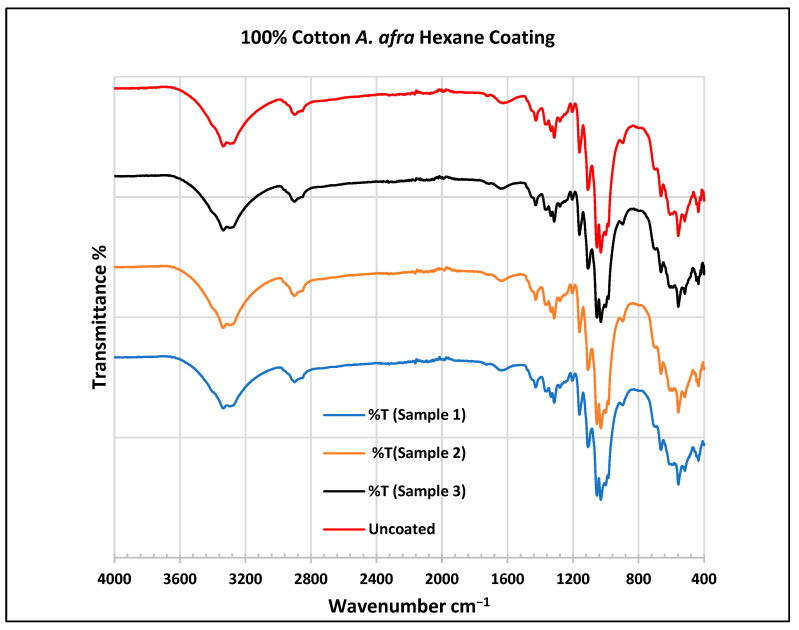
FTIR spectra of cotton fabrics uncoated (U) and coated (1–3) with *A. afra* hexane coating.

**Figure 11:**
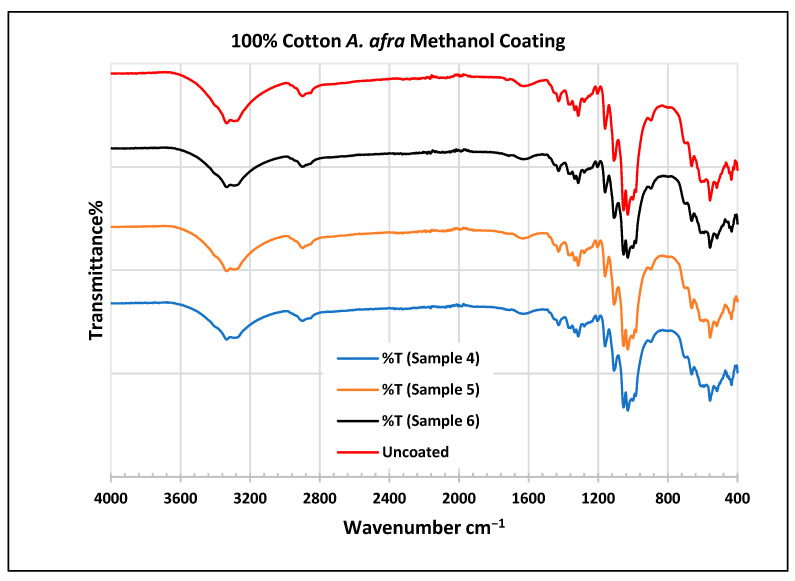
FTIR spectra of cotton fabrics uncoated (U) and coated (4–6) with *A. afra* methanol coating.

**Figure 12:**
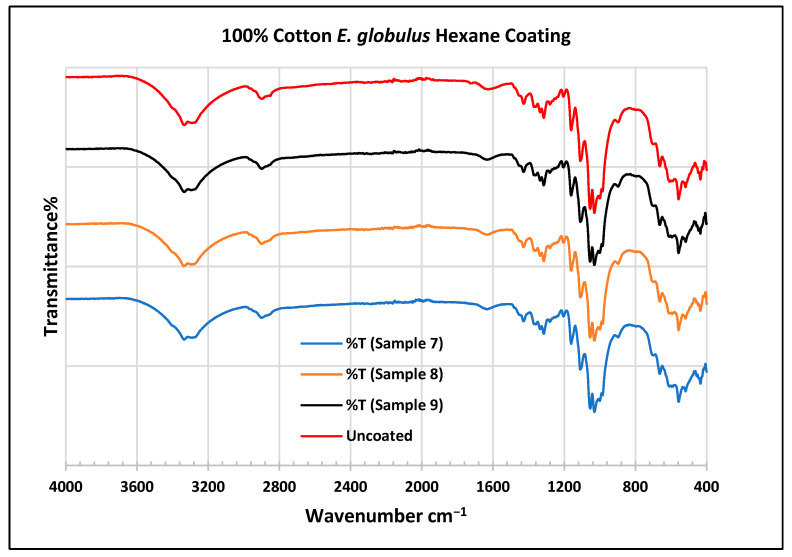
FTIR spectra of cotton fabrics uncoated (U) and coated (7–9) with *E. globulus* hexane coating.

**Figure 13:**
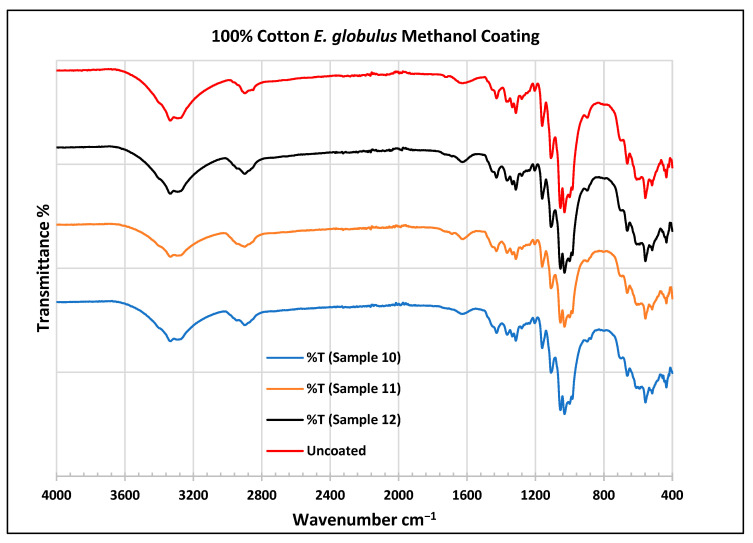
FTIR spectra of cotton fabrics uncoated (U) and coated (10–12) with *E. globulus* methanol coating.

To further validate that the fabric was modified by the extracts of *A. afra* and *E. globulus*, an FTIR study was conducted to understand the chemical attachment. The FTIR spectra of uncoated and coated polyester and cotton fabrics are presented in Figure 6, Figure 7, Figure 8, Figure 9, Figure 10, Figure 11, Figure 12 and Figure 13. The FTIR spectra were found to be like those reported by Sumantri et al. [32], Hayat et al. [33], and Nandiyanto et al. [34], but different from the FTIR spectra reported by Arockia et al. [35].

In the FTIR spectra analysis reported by Sumantri et al. [32], Hayat et al. [33], and Nandiyanto et al. [34], for the uncoated polyester fabric, the peak at 1715 cm^−1^ indicates the C=O group. The peaks in the region of 1502 cm^−1^ indicate vibrations, the aromatic ring peaks at 1473 cm^−1^ indicate the CH_2_ bending group and the peaks at 1411–1409 cm^−1^ indicate vibrations of an aromatic ring. The peaks at 1342–1340 cm^−1^ represent a CH_2_ wagging vibration of trans-co-formation, and the peak at 1097 cm^−1^ indicates CH in-plane bending modes of an aromatic ring. Strong peaks at 1243–1241 cm^−1^ indicate C-O stretching bonds. The peak at 967 cm^−1^ indicates trans-C-O stretching+ vibrations of the ester group and the peak at 872 cm^−1^ presents an aromatic ring. The peak at 724 cm^−1^ indicates ring CH out-of-plane bending + C=O out-of-plane bending groups.

For the coated *A. afra* hexane fabric, small peaks around 3427 cm^−1^ indicate -OH group bonds for samples 1–3. The peaks around 2958 cm^−1^, 2918 cm^−1^, and 2852 cm^−1^ indicate C-H stretching bonds from samples 1 to 3. Sample 3 shows strong peaks around this region whereas the peaks of samples 1–2 are weak but still indicate C-H stretching bonds for samples 1–3. The peaks from 1715 to 1713 cm^−1^ indicate the C=O stretching group for samples 1–3. The peaks around 1502 cm^−1^ indicate vibrations of an aromatic ring for samples 1–3 and the peaks at 1473–1471 cm^−1^ indicate CH_2_ bending bonds for samples 1–3. The peaks at 1410 cm^−1^ indicate an aromatic ring and the peak at 1340 cm^−1^ indicates a CH_2_ bending functional group for samples 1–3. The peaks between 1242 and 1232 cm^−1^ represent the C-O stretching group (ester) for samples 1–3. The peaks at 1096 cm^−1^ and in the range of 1019–1017 cm^−1^ indicate vibrations of an aromatic ring for samples 1–3. The peak range from 973 cm^−1^ to 965 cm^−1^ indicates C=C stretching trans and -C-H stretching out-of-plane bend for samples 1–3. The peaks ranging between 725 and 720 cm^−1^ indicate ring -C-H out-of-plane bending + C=O out-of-plane bending for samples 1–3.

In the FTIR analysis reported by Arockia et al. [35] for the coated polyester fabrics, from samples 1 to 3, the prominent peaks in the region of 2918 cm^−1^ indicate C-H symmetric stretching vibrations of CH_2_, which is an indication of the presence of lipids and protein. The vibrations at 1502 cm^−1^ indicate vibrations of an aromatic ring stretch indicating an aromatic compound, the peaks at 1473 cm^−1^ indicate the presence of a C=C-C aromatic compound, and the peaks at 1411–1409 cm ^−1^ indicate vibrations of an O-H bend indicating a phenol compound, as well as the peaks at 1342–1340 cm^−1^. The peak at 1097 cm^−1^ indicates the C-O stretch indicating the presence of cyclic ethers. Strong peaks at 1243–1241 cm^−1^ indicate C-O stretching bonds. The peaks at 967 cm^−1^ and 872 cm ^−1^ present P-O-C stretching, indicating the presence of aromatic phosphates. The peak at 724 cm^−1^ indicates the C-Cl stretch aliphatic chloro compound.

In the FTIR analysis reported by Sumantri et al. [32], Hayat et al. [33] and Nandiyanto et al. [34], the coated *A. afra* methanol fabrics from samples 4 to 6, the peak ranging from 2965 to 2851 cm^−1^ indicates the presence of C-H stretching bonds (aliphatic compounds C-H stretching in methyl and methylene groups). They are also identified between this range followed by peaks between 1470 and 1414 cm^−1^. The peaks at 1714 cm^−1^ indicate the presence of C=O stretching bonds. The small peaks at 1507 cm^−1^ indicate vibrations from an aromatic ring and the peaks at 1469 cm^−1^ CH_2_ indicate a bending functional group. The peaks at 1414 cm^−1^ indicate vibrations of an aromatic ring. The peaks at 1410 cm^−1^ indicate an aromatic ring. The peak at 1341 cm^−1^ indicates a CH_2_ bending functional group. The wide peaks at 1240 cm^−1^ indicate the C-O stretching group. The peaks at 1097 cm^−1^ and 1018 cm^−1^ indicate vibrations of an aromatic ring. The peaks in the range of 972 cm^−1^ and 872 cm^−1^ also indicate an aromatic ring. The peaks at 850 cm^−1^ indicate vibrations of an aromatic ring. The peak at 725 cm^−1^ indicates -C-H out-of-plane bending + C=O out-of-plane bending.

In the FTIR analysis reported by Arockia et al. [35], the coated *A. afra* methanol fabrics from samples 4 to 6, the prominent peaks ranging from 2965 to 2851 cm^−1^ indicate the presence of C-H stretching bonds (aliphatic compounds). The peaks at 1714 cm^−1^ indicate the presence of C=O, indicating a carbonyl compound. The small peaks in the range of 1507 cm^−1^ and 1469 cm^−1^ indicate C=C-C vibrations, indicating an aromatic compound. The peaks in the range of 1414–1410 cm^−1^ indicate vibrations of the O-H bend, indicating phenol compounds. The peak at 1341 cm^−1^ indicates O-H vibrations, indicating the presence of phenol compounds. The wide peaks at 1240 cm^−1^ indicate C-O stretching bonds. The peaks at 1097 cm^−1^ indicate the presence of the C-O stretching group, indicating the presence of cyclic ethers, and the peak at 1018 cm^−1^ indicates the presence of phosphate ions. The peaks in the range of 972 cm^−1^ and 850 cm^−1^ also indicate the P-O-C stretching group, indicating the presence of aromatic phosphates of an aromatic ring. The peak at 725 cm^−1^ indicates the C-Cl stretching group, indicating an aliphatic chloro compound.

In the FTIR analysis reported by Sumantri et al. [32], Hayat et al. [33], and Nandiyanto et al. [34], the *E. globulus* hexane-coated fabric from samples 7 to 9, the peaks at 2952 cm^−1^ indicate C-H stretching in methyl and methylene groups (aliphatic compounds), followed by peaks at 1471 and 725 cm^−1^. The peaks at 1714 cm^−1^ indicate a C=O stretching bond. There are peaks at 1506 cm^−1^, indicating that there are vibrations of an aromatic ring. The peaks around 1479 cm^−1^ indicate CH_2_ bending. The peaks at 1412–1402 cm^−1^ indicate an aromatic ring and the peaks at 1342 cm^−1^ indicate a CH_2_ bending functional group. The peaks between 1244 and 1237 cm^−1^ represent the C-O stretching group. The peaks at 1097 cm^−1^ indicate vibrations of an aromatic ring along with peaks in the range of 1017–972 cm^−1^ and peaks at 871 cm^−1^. The peaks ranging between 725 and 720 cm^−1^ indicate ring -C-H out-of-plane bending + C=O out-of-plane bending.

In the FTIR analysis reported by Arockia et al. [35], the *E. globulus* hexane-coated fabric from samples 7 to 9, the peaks at 2952 cm^−1^ indicate a C-H stretching group, indicating an alkane compound, followed by a peak at 1471 cm^−1^, which indicates an aromatic ring stretch showing an aromatic compound. The peak at 725 cm^−1^ indicates the C-Cl stretching group indicating an aliphatic chloro compound. The peaks at 1714 cm^−1^ indicate the C=O group, indicating the carbonyl group. There are peaks at band 1506 cm ^1^ indicating the C=C-C group, which indicates that there are vibrations of an aromatic ring. The peaks at 1479 cm^−1^ indicate an aromatic ring stretch C=C-C group, indicating an aromatic compound. The peak range 1412–1342 cm^−1^ indicates the O-H bend group, indicating phenol compounds. The peaks between 1244 and 1237 cm^−1^ represent the C-O stretching group. The peaks at 1097 cm^−1^ indicate the presence of the C-O stretching group, indicating the presence of cyclic ethers and the peak at 1018 cm^−1^ indicates the presence of phosphate ions. The peaks in the range of 972 cm^−1^ and 850 cm^−1^ also indicate the P-O-C stretching group, indicating the presence of aromatic phosphates of an aromatic ring. The peak at 725 cm^−1^ indicates the C-Cl stretching group indicating an aliphatic chloro compound.

In the FTIR analysis reported by Sumantri et al. [32], Hayat et al. [33], and Nandiyanto et al. (2019) [32,33,34], the *E. globulus* methanol-coated fabric from samples 10 to 12, the peaks around 2935 cm^−1^ indicate C-H stretching in methyl and methylene groups. The peaks in the range of 1714–1710 cm^−1^ indicate C=O stretching bonds. The peak 1411 cm^−1^ indicates an aromatic ring and the peak 1342 cm ^−1^ indicates a CH_2_ bending functional group. The peak range 1247–1237 cm^−1^ indicates the C-O stretching group. The peaks at 1092 cm^−1^ indicate vibrations of an aromatic ring. The peaks at 1018 cm^−1^ and 873 cm^−1^ indicate vibrations of an aromatic ring. The peak range of 847–735 cm^−1^ indicates vibrations of aromatic rings. The peaks at 725–722 cm^−1^ indicate CH out-of-plane bending + C=O out-of-plane bending.

In the FTIR analysis reported by Arockia et al. [35], the *E. globulus* methanol-coated fabric from samples 10 to 12, the peaks around 2935 cm^−1^ indicate asymmetric stretching of -CH(CH_2_) vibration indicating the presence of lipids and protein compounds. The peaks in the range of 1714–1710 cm^−1^ indicate C=O stretching bonds, indicating carbonyl compounds. The peaks in the range of 1412–1342 cm^−1^ indicate the O-H bend group, indicating phenol compounds. The peaks between 1247 and 1237 cm^−1^ represent the C-O stretching group. The peaks at 1097 cm^−1^ indicate the presence of the C-O stretching group, indicating the presence of cyclic ethers, and the peak at 1018 cm^−1^ indicates the presence of phosphate ions. The peaks in the range of 972 cm^−1^ and 850 cm^−1^ also indicate the P-O-C stretching group, indicating the presence of aromatic phosphates of an aromatic ring. The peak in the range of 725–722 cm^−1^ indicates the C-Cl stretching group, indicating an aliphatic chloro compound.

In the FTIR analysis reported by Sumantri et al. [32], Hayat et al. [33] and Nandiyanto et al. [34], the uncoated cotton, the peaks for cotton fabrics are found at around 3326 cm^−1^ corresponding to O-H stretching and in the 2897 cm^−1^ region for C-H stretching in methyl and methylene groups. The peak at 1611 cm^−1^ indicates the C=O carbonyl group. The peaks at 1417 cm^−1^ indicate vibrations of an aromatic ring. A peak at 1311 cm^−1^ is associated with the O-H group and peaks at 1120 cm^−1^, 1066 cm^−1^, and 1120–1029 cm^−1^ indicate C-O stretching. The peaks at 895 cm^−1^ indicate the C=C group and the peaks at 680–629 cm^−1^ indicate the alkyne C-H bend.

For the cotton, *A. afra* hexane-coated fabric from samples 1 to 3, the peaks at 3323 cm^−1^ indicate O-H stretching. A small wide absorption peak at 2889 cm^−1^ indicates C-H stretching in methyl and methylene groups. The peak at 1429 cm^−1^ indicates the bending of the CH group and peaks around 1316 cm^−1^ are associated with CH_2_ groups. The peaks between 1129 and 1119 cm^−1^ indicate C-O stretching of trans conformers. The peaks at 1109 cm^−1^ indicate a C-O stretch is associated with CH_2_ groups—cyclohexane vibrations at 1057 cm^−1^ and 1000 cm^−1^. The peaks from 667 to 620 cm^−1^ indicate an alkyne C-H bend. For the cotton, *A. afra* hexane-coated fabric from samples 1–3, the peaks at 3323 cm^−1^ indicate O-H stretching. A small wide absorption peak at 2889 cm^−1^ indicates C-H stretching in methyl and methylene groups. A peak at 1429 cm^−1^ indicates the bending of the CH group and the peak at 1316 cm^−1^ is associated with CH_2_ groups. The peaks between 1129 and 1119 cm^−1^ indicate C-O stretching of trans conformers. The peaks at 1109 cm^−1^ indicate a C-O stretch is associated with CH_2_ groups—cyclohexane vibrations at 1057 cm^−1^ and 1000 cm^−1^. The peaks from 667 to 620 cm^−1^ indicate an alkyne C-H bend.

In the FTIR analysis reported by Arockia et al. [35], the peaks for cotton fabrics are found at around 3326 cm^−1^ corresponding to the O-H stretching group, indicating polyhydroxy compounds. The peak at 1611 cm^−1^ indicates the C=O stretching vibrations, indicating a ketone group. A peak at 1311 cm^−1^ is associated with the O-H group indicating a phenol compound, and peaks at 1120 cm^−1^ indicate a C-O stretch, indicating a cyclic ether compound. The peaks at 895 cm^−1^ indicate aromatic phosphates (P-O-C group) and the peaks at 680–629 cm^−1^ indicate the C-Br group, indicating the presence of aliphatic bromo compounds.

In the FTIR analysis reported by Sumantri et al. [32], Hayat et al. [33], and Nandiyanto et al. [34], the cotton *A. afra* methanol-coated fabric from samples 4 to 6, the peaks between 3340 and 3268 cm^−1^ indicate the -OH group. The peaks in the range of 2897–2823 cm^−1^ indicate C-H stretching in methyl and methylene groups. The peaks at 1425 cm^−1^ indicate vibrations of an aromatic ring. The peaks at 1315 cm^−1^ are associated with the –CH_2_ group. The peaks at 1163–1123 cm^−1^ are associated with C-O stretching as well as peaks at 1105–1068 cm^−1^. The peaks at 1000 cm^−1^ indicate vibrations of an aromatic ring. The peaks at 681–631 cm^−1^ indicate an alkyne C-H bending group.

In the FTIR analysis reported by Arockia et al. [35], the cotton *A. afra* methanol-coated fabric from samples 4 to 6, the peaks between 3340 and 3268 cm^−1^ indicate the O-H stretching group, which shows a polyhydroxy compound. The peaks at 2897–2823 cm^−1^ indicate C-H stretching in methyl and methylene groups. The peaks at 1425 cm^−1^ indicate vibrations of an aromatic ring. The peaks at 1315 cm^−1^ are associated with the O-H bending group, which indicates the presence of a phenol group. The peaks in the range 1105–1000 cm^−1^ indicate a phosphate ion group, which indicates the presence of phosphate compounds of an aromatic ring. The peaks at 681–631 cm^−1^ indicate C-Br stretching (aliphatic bromo compounds).

In the FTIR analysis reported by Sumantri et al. [32], Hayat et al. [33], and Nandiyanto et al. [34], the cotton *E. globulus* hexane-coated fabric from samples 7 to 9, the peaks ranging between 3333 and 3254 cm^−1^ indicate O-H stretching. The peaks at 2879–2823 cm^−1^ indicate C-H stretching in methyl and methylene groups. Small peaks ranging from 1636 to 1626 cm^−1^ indicate asymmetrical stretching vibration of the COO- bond. The peaks at 1428 cm^−1^ indicate the bending of the CH group and the peaks at 1372 cm^−1^ indicate the CH_2_ wagging vibration of the gauche conformation. The peaks in the range of 1317–1307 cm^−1^ indicate asymmetric stretching of the C-O-C group. The peaks 1124–1068 cm^−1^ indicate C-O stretching of trans conformers. The peaks at 1000 cm^−1^ indicate vibrations of an aromatic ring. The peaks at 622–612 cm^−1^ indicate an alkyne C-H bend.

In the FTIR analysis reported by Arockia et al. [35], the cotton *E. globulus* hexane-coated fabric from samples 7 to 9, the peaks ranging between 3333 and 3254 cm^−1^ indicate the O-H stretching group, which indicates a polyhydroxy compound. The small peaks ranging from 1636 to 1626 cm^−1^ indicate C-O stretching, which indicates the ketone compound. The peaks in the range of 1372–1307 cm^−1^ indicate an O-H bending group, which indicates a phenol compound. The peaks at 1000 cm^−1^ indicate vibrations of the phosphate ion group, which indicates the presence of a phosphate compound. The peaks in the range of 622–612 cm^−1^ indicate an alkyne C-Br vibration, which indicates an aliphatic bromo compound.

In the FTIR analysis reported by Sumantri et al. [32], Hayat et al. [33], and Nandiyanto et al. [34], the cotton *E. globulus* methanol-coated fabric from samples 10 to 12, the peaks around 3332–3270 cm^−1^ indicate the -OH cm^−1^ group and the peaks around 2935 cm^−1^ were observed and indicate C-H stretching in the methyl and methylene groups. The peaks in the range of 1714–1710 cm^−1^ indicate C=O stretching bonds. The peaks at 1411 cm^−1^ indicate an aromatic ring. The peak at 1342 cm^−1^ indicates a CH_2_ bending functional group. The peak range of 1247–1237 cm^−1^ indicates the C-O stretching group. The peaks at 1092 cm^−1^ indicate vibrations of an aromatic ring. The peaks at 1018 cm^−1^ and 873 cm^−1^ indicate vibrations of the aromatic ring. The peaks in the range of 847–735 cm^−1^ indicate vibrations of aromatic rings. The peaks in the range of 725–722 cm^−1^ indicate CH out-of-plane bending + C=O out-of-plane bending.

In the FTIR analysis reported by Arockia et al. [35], the cotton *E. globulus* methanol-coated fabric from samples 10 to 12, the peaks around 3332–3270 cm^−1^ indicate an O-H stretching group, which indicates a polyhydroxy compound, and the peaks around 2935 cm^−1^ were observed and indicate asymmetric stretching of -CH(CH_2_) vibration, indicating a saturated aliphatic compound. The peaks in the range of 1714–1710 cm^−1^ indicate the C=O stretch indicating the carbonyl group. The peaks in the range of 1412–1342 cm^−1^ indicate the O-H bend group, indicating phenol compounds. The peaks between 1247 and 1237 cm^−1^ represent the C-O stretching group. The peaks at 1097 cm^−1^ indicate the presence of the C-O stretching group, indicating the presence of cyclic ethers and the peaks at 1018 cm^−1^ indicate the presence of phosphate ions. The peaks in the range of 972 cm^−1^ and 850 cm^−1^ also indicate the P-O-C stretching group, indicating the presence of aromatic phosphates of an aromatic ring. The peak in the range of 725–722 cm^−1^ indicates the C-Cl stretching group, which indicates an aliphatic chloro compound.

The FTIR spectrum results reveal that the methanol and n-hexane extract functional groups exhibit absorption signals across multiple wavenumber ranges. These functional groups contain chemical bonds that may contribute to their antimicrobial properties. For instance, the presence of alcohols and phenols (O-H), polyhydroxy compounds (O-H stretching in the extracts), carboxylic acids (C-O stretching), and methyl and aldehyde groups (stretching of C-H bonds) may disrupt cell membranes or inhibit bacterial enzymes, thus potentially enhancing the extracts’ antimicrobial activity. Moreover, alkenes (C=C stretching) and aromatics (C-C stretching group) may induce oxidative stress in bacterial cells, contributing to the extracts’ anti-inflammatory and antimicrobial properties. Additionally, CH_2_ methylene groups and aliphatic compounds (C-H stretching), aliphatic bromo compounds (C-Br), and P-O-C stretch (aromatic phosphates) may also be involved in the biological activity of the extracts. Polyhydroxy compounds (O-H stretch) may contribute to the extracts’ anti-inflammatory and immunomodulatory properties, in addition to their antimicrobial activity. It is worth noting that the chemical composition of the extracts may vary depending on factors such as geographical location, climate conditions, drying methods, population variability, and extraction solvents.

Overall, based on the FTIR spectrum results, it appears that the methanol and n-hexane extracts of *Artemisia afra* and *Eucalyptus globulus* contain a complex mixture of bioactive compounds with potential antimicrobial properties.

## 4. Conclusions

In this study, we explored the properties of two medicinal plants, *Eucalyptus globulus* and *Artemisia afra.* The research encompassed the yield output, phytochemical screening compounds, biological activities against selected test strains before and after textile coating, minimum inhibitory concentration, zone of inhibition, durability to laundering, SEM analysis of the coated fabric, and characterization of the coated fabrics. The results indicated that E. globulus methanol extraction yielded the highest at 22.76% (±0.61%), followed by hexane extraction at 3.98% (±0.06%). *A. afra* methanol extraction showed a yield of 9.22% (±0.40%), followed by hexane extraction at 2.19% (±0.28%). Methanolic extracts of both plants exhibited higher yields compared with hexanoic extracts, with *A. afra* producing the lowest yield. Phytochemical screening revealed the presence of various secondary metabolic compounds, such as phenols, quinones, terpenoids, flavonoids, saponins, steroids, and tannins, in both plants. Notably, methanol extracts displayed positive results for the studied phytochemicals. Antimicrobial activity was observed against *S. aureus*, with *E. globulus* hexanoic extracts being the most effective. However, *Escherichia coli* ATCC 25,922 showed resistance to plant extracts, with *A. afra* methanolic extracts exhibiting slight activity.

Minimum inhibitory concentration (MIC) values ranged from 5 μg/mL to above 500 μg/mL, and complete inhibition of cultures did not occur. Laundering significantly reduced antimicrobial activity, possibly due to inactivation of active compounds.

Coating fabrics with *E. globulus* methanolic extract exhibited antibacterial activity, as indicated by larger zones of inhibition compared with untreated samples. SEM analysis revealed clear particles on coated fabric surfaces.

After one washing cycle, no activity was observed against *S. aureus* and *E. coli*. FTIR spectrum results indicated the presence of bioactive compounds in the extracts, suggesting potential antimicrobial properties. Further research on plant-based antimicrobial agents on textile substrates and green extraction techniques is warranted for extended longevity and durability to laundering.

## Figures and Tables

**Table 1:** Extraction conditions.

No.	Parameter	PUAE Extraction
**1**	Sample size (g)	10 g
**2**	Extraction solvent	n-hexane and methanol
**3**	Solvent volume (mL)	100 mL
**4**	Temperature (°C)	40 °C
**5**	Time (min)	20 min

**Table 2:** The average yield percentage of the plant extracts obtained by the PUAE method.

$\mathrm{Average}\;\mathrm{Yield}\;\%=\frac{Weight\;of\;the\;extract\;after\;solvent\;removal}{Dry\;weight\;of\;plant\;sample}\times 100\%$				
	*A. afra* Hexane Extract (g)	*A. afra* Methanol Extract (g)	*E. globulus* Hexane Extract (g)	*E. globulus* Methanol Extract (g)
	0.259	0.979	0.390	2.233
	0.198	0.899	0.403	2.234
	0.202	0.888	0.400	2.362
Avg. Yield %	2.192	9.221	3.979	22.762
**SD%**	0.280	0.403	0.055	0.607

Data are means of three replicates (*n* = 3) ± standard error.

**Table 3:** Phytochemical compounds of *A. afra* and *E. globulus* extracts.

Plant	*Artemisia afra*		*Eucalyptus globulus*	
Solvent	Methanol	n-Hexane	Methanol	n-Hexane
Phytochemicals				
Phenol	+	+	+	+
Flavonoids	+	+	−	+
Quinones	+	+	+	+
Tannins	+	−	+	−
Saponins	+	−	+	−
Terpenoids	+	−	+	+
Steroids	+	+	+	+

−: absence, +: presence.

**Table 4:** Summary of the antibacterial activity detected against two ATCC test strains when a concentration range of 5–500 μg/mL was evaluated in a 96-well bioactivity assay. *S. aureus*: *Staphylococcus aureus*; *E. coli*: *Escherichia coli*; A. a: *Artemisia afra*; E. g: *Eucalyptus globulus*; H: hexane; and M: methanol.

Sample No. Plant Extract	MIC Values (µg/mL)	
	Microorganisms	
	Gram-Negative Bacteria *E. coli* ATCC 25922 (Tryptic Soy Broth)	Gram-Positive Bacteria *S. aureus* subsp. *Aureus* ATCC 33,591 (Nutrient Broth)
1 A. aH	50–500 μg/mL	5–25 μg/mL; 250–500 μg/mL
2 A. aH	10–500 μg/mL	125–500 μg/mL
3 A. aH	None	50–500 μg/mL
4 A. aM	5–500 μg/mL	5–500 μg/mL
5 A. aM	5–500 μg/mL	5 μg/mL; 125–500 μg/mL
6 A. aM	10–500 μg/mL	100–500 μg/mL
7 E. gH	5–50 μg/mL *	None
8 E. gH	250–500 μg/mL	None
9 E. gH	100–500 μg/mL	None
10 E. gM	None	None
11 E. gM	None	250–500 μg/mL
12 E. gM	None	None

Data are means of three replicates (n = 3) ± standard error. * If activity is only observed at lower concentrations, it usually means that a compound inhibitor is being diluted out, resulting in activity only being observed at the lower (more dilute) sample range. Samples 1–12 were performed in triplicate.

**Table 5:** Summary of the bioactivity detected for material samples infused with plant extracts evaluated against *E. coli* ATCC 25,922 and *S. aureus* subsp. *Aureus* ATCC 33591.

Sample No. Plant Extract	Material Type	Disk Diameter	Zone Diameter	Zone of Inhibition
10 E. gMC	Light blue/grey cotton	20 mm	23 mm	101.32 mm^2^
10 E. gMP	Turquoise polyester	20 mm	22 mm	65.97 mm^2^
11 E. gMC	Light blue/grey cotton	20 mm	27 mm	258.40 mm^2^
11 E. gMP	Turquoise polyester	19 mm	22 mm	96.60 mm^2^
12 E. gMC	Light blue/grey cotton	20 mm	25 mm	176.71 mm^2^

Data are means of three replicates (n = 3) ± standard error. Sample 10 E. gMC: light blue grey *E. globulus* methanolic-coated cotton fabric; 10 E. gMP: turquoise *E. globulus* methanolic-coated polyester fabric; 11 E. gMC: light blue grey methanolic cotton-coated fabric; 11 E. gMP: turquoise *E. globulus* methanolic-coated polyester fabric; and 12 E. gMC: light grey/blue methanolic-coated cotton fabric. Samples 1–12 were performed in triplicate on the light blue/grey cotton and turquoise polyester fabrics.

## Data Availability

Data is contained within the article.

## Abbreviations

ISO	International Standards Organization
PUAEs	Pulsed Ultrasonic Assisted Extractions
PPE	Personal Protective Equipment
MIC	Minimum inhibitory concentration
DMSO	Dimethyl sulfoxide
ZOI	Zone of inhibition
UV	Ultraviolet
COVID-19	Coronavirus Disease of 2019
*w*/*v*	Weight-to-volume ratio
FTIR	Fourier-transform infrared spectroscopy
MTT	(3-(4,5-Dimethylthiazol-2-yl)-2,5-Diphenyltetrazolium Bromide)
*E. coli*	*Escherichia coli*
*S. aureus*	*Staphylococcus aureus*
*S. pyogenes*	*Streptococcus pyogenes*
*H. influenzae*	*Hemophilus influenzae*
AATCC	American Association of Textile Chemists and Colorists
PPL	Priority Pathogen List
WHO	World Health Organization
